# Cell Size Decrease and Altered Size Structure of Phytoplankton Constrain Ecosystem Functioning in the Middle Danube River Over Multiple Decades

**DOI:** 10.1007/s10021-019-00467-6

**Published:** 2019-12-03

**Authors:** András Abonyi, Keve Tihamér Kiss, András Hidas, Gábor Borics, Gábor Várbíró, Éva Ács

**Affiliations:** 1 Centre for Ecological Research, Institute of Ecology and Botany, Alkotmány u 2-4, Vácrátót, 2163 Hungary; 2 Centre for Ecological Research, Danube Research Institute, Karolina u 29, Budapest, 1113 Hungary; 3 WasserCluster Lunz, Biologische Station GmbH, Dr. Carl Kupelwieser Promenade 5, 3293 Lunz am See, Austria; 4 Doctoral School of Environmental Sciences, Eötvös Loránd University, Pázmány Péter sétány 1/A, Budapest, 1117 Hungary; 5 Centre for Ecological Research, GINOP Sustainable Ecosystems Group, Hungarian Academy of Sciences, Klebelsberg Kuno u 3, Tihany, 8237 Hungary; 6 Faculty of Water Sciences, National University of Public Service, Bajcsy-Zsilinszky u 12-14, Baja, 6500 Hungary

**Keywords:** Body size, Community response, Ecosystem functioning, Ectothermic aquatic organisms, Global warming, Human impacts, Large rivers, Potamoplankton

## Abstract

Reduced body size is among the universal ecological responses to global warming. Our knowledge on how altered body size affects ecosystem functioning in ectothermic aquatic organisms is still limited. We analysed trends in the cell size structure of phytoplankton in the middle Danube River over a 34-year period at multiple levels: (1) average cell size of assemblages (ACS), (2) within the centric diatom community and (3) in the dominant centric diatom taxon: *Stephanodiscus*. We asked whether global warming and human impacts affected the average cell size of phytoplankton. Also, whether the altered size structure affected how chlorophyll-*a*, as an ecosystem functioning measure, relates to the ACS of phytoplankton. The cell size of phytoplankton decreased significantly at all organisation levels, and the assemblages became more dispersed in cell size over time. Environmental variables related to global warming and human impacts affected the ACS of phytoplankton significantly. The relationship between chlorophyll-*a* and the ACS of phytoplankton shifted from negative linear to broad and then narrow hump shape over time. Longer water residence time, warming and decline in nutrients and suspended solids decrease the ACS of phytoplankton in the middle Danube and expectedly in other large rivers. Our results suggest that cell size decrease in phytoplankton, especially of centric diatoms, constrains planktic algal biomass production in large rivers, independently of algal density. Such cell size decrease may also affect higher trophic levels and enhance the more frequent occurrence of “clear-water” plankton in large, human-impacted rivers under global change.

**Electronic supplementary material:**

The online version of this article (10.1007/s10021-019-00467-6) contains supplementary material, which is available to authorized users.

## Highlights

- The average cell size of phytoplankton decreases in the middle Danube River.
- Altered size structure originates from both compositional shift and adaptation.
- The altered size structure constrains planktic algal biomass production.

## Introduction

Body size is a key ecological trait that affects fitness via growth and reproduction (effect trait) and responds to alterations in the environment (response trait) (Hooper and others 2005). Accordingly, body size is a useful ecological indicator of—among others—global warming- and human-induced changes in the environment. Global warming increases air and water temperature that alter body size via metabolic rates of organisms (Gillooly and others 2001). Alteration in body size, in turn, is expected to affect ecosystem functioning in experimental communities (Yvon-Durocher and others 2011). Reduced body size is among the universal ecological responses to global warming (Daufresne and others 2009), whereas body size reduction is expectedly larger in aquatic than in terrestrial systems (Forster and others 2012). Long-term changes in cell size of aquatic ectothermic organisms like phytoplankton have extensively been studied (Finkel and others 2005; Smol and others 2005; Mousing and others 2014). However, our knowledge on how the altered cell size structure of communities affects ecosystem functioning, especially in river phytoplankton assemblages, is still limited.

According to global climate change scenarios, the air temperature will continue to increase on average, as well as the water temperature of oceans, lakes and rivers that will affect aquatic biota further (IPCC 2007). Global warming enhances thermal stratification in marine and freshwater systems (DiNezio and others 2009; Kraemer and others 2015) favouring small-sized phytoplankton (Bopp and others 2005; Winder and others 2009). However, the effect of lowering water discharge, or the more frequent occurrence of lower water discharge due to climate change in large rivers, is unknown (while might be similar in effect as the enhanced water retention time and stronger thermal stratification in lakes). In the ocean and freshwaters, enhanced stratification leads to decline in nutrients in the upper strata (Schmittner 2005; Winder and others 2009). Nutrient-deficient environments then favour small-sized phytoplankton individuals due to their more efficient nutrient uptake (Lewis 1976; Finkel and others 2010). Oligotrophic conditions are expanding in aquatic ecosystems and are coupled with the dominance of small-sized phytoplankton (Irwin and Oliver 2009). In large rivers, especially in W-Europe, long-term decline in nutrients is now a general trend (Ibáñez and Peñuelas 2019). That could, therefore, affect the cell size structure of large river phytoplankton in a very similar way observed in lake and marine environments. Such long-term response in natural large river phytoplankton, however, is mostly unknown.

Human impacts such as damming, eutrophication, decreased water discharge due to irrigation all affect large rivers, defined as the Anthropocene syndromes (Meybeck 2003). Eutrophication has been mitigated in several European rivers by effective regulatory actions following the implementation of the European Water Framework Directive (WFD 2000). In response, re-oligotrophication has recently been reported in several large European rivers (Hardenbicker and others 2014; Minaudo and others 2015; Abonyi and others 2018; Ibáñez and Peñuelas 2019). In the middle section of the Danube River, the concentration of nutrients declined in response to enhanced sewage control (Istvánovics and Honti 2012) and increased water retention time due to damming at the upper river sections (Kiss 1994; Abonyi and others 2018). At the same time, the water temperature increased gradually. Accordingly, phytoplankton of the middle Danube River is an excellent natural system to study the ecological response of lotic primary producers to global warming and human impacts. Also, how the response of phytoplankton affected ecosystem functioning at long temporal scale.

The functional community composition of the middle Danube River phytoplankton responded to long-term alterations in the environment (Abonyi and others 2018). Chlorophyll-*a* as a proxy of planktic algal biomass declined over time. The taxonomic richness of phytoplankton decreased as well, whereas functional diversity of phytoplankton increased significantly (opt. cit.). Although it is obvious that smaller phytoplankton taxa now do occur more frequently in the middle Danube (opt. cit.), we do not know whether a long-term decrease in cell size also occurs within specific taxonomic units. Notably, whether centric diatoms as the best-adapted and therefore the most productive algal group of large rivers also decreased in cell size.

Our question has primary importance because the lifestyle of phytoplankton depends on the entrainment of cells in water motion (Reynolds 2006). The sinking velocity of phytoplankton scales linearly with turbulent velocity within the micro-phytoplankton size range (Reynolds 1997). Body size is one of the organismic properties open to evolution, and therefore, through which phytoplankton can adapt to environmental constraints (opt. cit.). Long-term water temperature increase together with the more frequent occurrence of lower water discharge in the Danube River affects the viscosity of water and the extent of turbulent motion in the water mass. We, therefore, expect alterations in the mechanisms of entrainment and disentrainment of phytoplankton in the middle Danube River over time. That is, altered mechanisms selecting for the appropriate body size are either within the phenotypic plasticity of taxa (adaptation), or by species replacement (compositional changes).

We hypothesise that the phytoplankton cell size in the middle Danube River decreased over time, resulting in lower average cell size (ACS) based on the total algal biovolume to the total algal abundance ratio. We also hypothesise that the cell size of the centric diatom community decreased, including the core centric diatom families, Stephanodiscaceae–Thalassiosiraceae (*Stephanodiscus* hereafter).

Using harmonised time intervals, we analyse time trends in cell size (biovolume) at the aforementioned organisation levels of the Danube phytoplankton.

Moreover, we expect that the ACS of phytoplankton represents an assemblage-level response to long-term alterations in environmental conditions. Here, using generalised additive models (GAMs), we predict the ACS of phytoplankton from environmental variables related to global warming (water temperature, water discharge) and human impacts (total suspended solids, orthophosphate-P).

Finally, we are interested in whether the altered cell size structure of phytoplankton affected the relationship between chlorophyll-*a*—as an independent measure of algal biomass—and the ACS of phytoplankton over time. Because primary production is affected by both light and nutrients, chlorophyll-*a* is a proxy for combined resource use (Marañón 2015). Accordingly, it can be used as an ecosystem functioning measure (Ptacnik and others 2008). Using GAM, we first model chlorophyll-*a* from algal abundance and then use the ACS of phytoplankton as an additional predictor. We expect that the ACS of phytoplankton represents reliable ecological information on top of algal abundance; therefore, it enhances our ability in predicting ecosystem functioning of phytoplankton in the middle section of the Danube River.

## Materials and methods

### Location of the Sampling Station (Göd, N-Budapest, Hungary)

The long-term phytoplankton monitoring station of the Centre for Ecological Research is located at Göd, approximately 20 km upstream from Budapest (1668 r.km, distance from the mouth), capital of Hungary. Detailed information about the sampling location can be found in Duleba and others (2014), Tóth and Bódis (2015), and Abonyi and others (2018).

### Phytoplankton Analysis

Phytoplankton samples were taken once a week from the middle of the thalweg between 1979 and 2012 and fixed with acetic Lugol’s solution. Microscopic count and identification of phytoplankton were carried out using the same approach (Utermöhl 1958) and by the same person (Keve T. Kiss, second author) over the entire period. Algal biovolume (except centric diatoms, see below) was calculated from characteristic geometrical forms (Hillebrand and others 1999) using the average cell size of at least 20 individuals from populations in the middle section of the Danube. Biovolume of taxa, therefore, did not follow potential long-term cell size changes in all individual species. Biomass was expressed as fresh weight biovolume assuming a density of 1. Our phytoplankton data set has been analysed for consistency (see Abonyi and others 2018; Supplement material 2) along with pitfalls occurring potentially in such long-term data sets (Straile and others 2013).

The cell size of centric diatoms has not been measured in each sample for the entire study period. Because the calculation of biovolume was not required before its implementation into ecological status monitoring of surface waters in Europe (WFD 2000), in the early years of the monitoring, only algal density was counted. Here, from stored samples, aliquot volumes were mixed within each season of each year. This enabled us to measure cell size and calculate a seasonal median biovolume of centric diatoms for each season. The same median biovolume value of centrics was then used to calculate the total phytoplankton biovolume for all samples of the same season. Diatom valves have been cleaned using the hydrochloric acid and hydrogen peroxide approach (CEN 2014). From aliquot volumes, the diameter of the first 100 centric diatom valves has been measured. This resulted in more than 12,000 measurements covering the 34 years, which number is equal to a monthly monitoring that would have measured 30 individuals in each phytoplankton sample. Missing samples as well as those with less than 50 diameter measurements have been excluded from data analyses.

Cell height is highly variable among centric diatom taxa, but is required for biovolume calculations. We calculated the cell height from cell diameter using a random ratio between 30% (extreme flattened cylindrical form, for example, *Discostella* spp.) and 80% (extreme cuboid cylindrical form, for example, *Cyclotella meneghiniana*) (function runif (diameter, min = diameter × 0.3, max = diameter × 0.8). This allowed us to analyse time trends (see below) in the cell size structure without considering the taxonomy of centrics. For time-trend analysis in the biovolume of the Stephanodiscaceae–Thalassiosiraceae families (*Stephanodiscus*), we calculated the biovolume using the fix factor of 60% between cell height and cell diameter (an average cuboid-like cylindrical form).

The average cell size (ACS) of phytoplankton was calculated based on the total phytoplankton biovolume to the total algal abundance (number of individuals) ratio, which gives a reliable response to alterations in environmental drivers (Sommer and others 2017). The ACS of phytoplankton, therefore, allows us to follow alterations in the cell size (biovolume/body size) structure of phytoplankton independently of the total biovolume (biomass) and density of assemblages.

### Data Selection and Statistical Analyses

Hydrological data (water discharge) were provided by the General Directorate of Water Management (Budapest). Water temperature was measured in situ, total suspended solids (TSSs) gravimetrically, nutrients (nitrate-N, nitrite-N, ammonium-N, orthophosphate-P) and chlorophyll-*a* using spectrophotometric approaches (see Duleba and others 2014). Environmental variables that affected the average cell size of phytoplankton, as well as chlorophyll-*a*, have all been selected by generalised additive model (GAM) (Wood 2011).

To reveal time trends in the ACS of phytoplankton, in the cell size of the centric diatom community and of *Stephanodiscus*, the Seasonal Mann–Kendall—“SMK” (Hirsch and Slack 1984), and the Mann–Kendall—“MK”—trend tests were used in the *Kendall* R package (McLeod 2011). The ACS of phytoplankton was analysed based on mean data in each month, whereas the cell sizes of the centric diatom community and of *Stephanodiscus* were analysed based on median values in each season. Temporal aggregation of data resulted in harmonised time intervals required for time-trend analysis, increased the significance level of trends and reduced temporal autocorrelation (McLeod 2011). For all Mann–Kendall trend tests, we used the block bootstrap approach in the *boot* R package (Davison and Hinkley 1997; Canty and Ripley 2017) to perform bootstrap confidence interval calculations using 10,000 bootstrap replicates at 99% confidence interval (CI).

Based on the entire weekly data set (with the same median cell biovolume data for centric diatoms in each season), we modelled the ACS of phytoplankton from environmental variables using generalised additive model (GAM) (Wood 2011), selected according to the Akaike’s information criterion (AIC). Here, we tested whether environmental variables with long-term changes (see Abonyi and others 2018) affected the ACS of phytoplankton significantly, as an assemblage-level functional response trait. The model included ln-transformed environmental data except for water temperature, which was sqrt-transformed; year and month (time) were used as random factors (bs=“re” in gam in package *mgcv*; Wood 2011).

Based on the entire weekly data set (with the same median cell biovolume data for centric diatoms in each season), we modelled chlorophyll-*a* from (1) algal abundance (ABU) and (2) the average cell size of phytoplankton (ACS) using generalised additive models (GAMs) (Wood 2011). In a preliminary analysis (see Supplement 2), the ACS of phytoplankton predicted chlorophyll-*a* in different ways between three distinct periods: before 1990 (P1), between 1990 and 2000 (P2) and after 2000 (P3). Accordingly, we ran separate GAM models in each period. Here, we tested whether alterations in the ACS of phytoplankton (as an assemblage-level response trait) affected the relationship between planktic algal biomass (independent chlorophyll-*a* measure of the count data) and the ACS of phytoplankton in the three periods.

Furthermore, we tested whether the ACS of phytoplankton added valuable ecological information in predicting chlorophyll-*a* on top of algal density. Here, we tested whether bootstrapped coefficients of determinations (*R*^2^) and AIC values of GAMs differed significantly between the two models: (1) ABU predicting chlorophyll-*a* and (2) ABU + ACS predicting chlorophyll-*a* (*boot* in R (Davison and Hinkley 1997; Canty and Ripley 2017) with 999 replicates). In GAMs, year and month (time) were used as random factors (bs = “re” in gam in package *mgcv*; Wood 2011). The bootstrapped coefficients of determinations and AIC values were compared by Wilcoxon rank-sum tests.

All data analyses and visualisations were performed in R (R Core Team 2018).

## Results

### Long-Term Trends in Phytoplankton Cell Size in the Middle Danube Section

**Figure 1:**
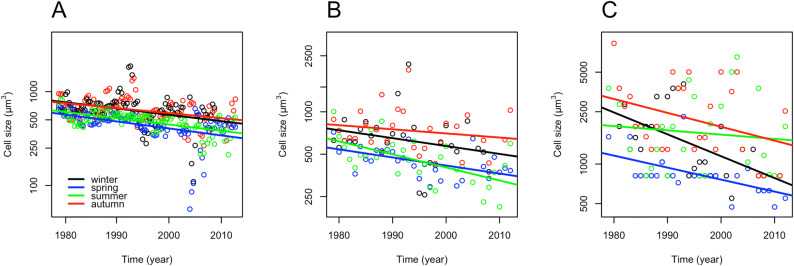
Seasonal long-term linear trends (1979–2012) **A** in the average cell size of phytoplankton assemblages; **B** in the cell size of the centric diatom community; and **C** in the cell size of the Stephanodiscaceae–Thalassiosiraceae families in the middle Danube section (Göd, N-Budapest, Hungary). Data are based on mean values in each month (**A**), and median values in each season (**B**,** C**).

The average cell size of phytoplankton decreased significantly in the pooled data set of all seasons (Seasonal MK, tau: − 0.33, *p* < 0.001). In individual seasons (Figure 1A), it decreased significantly in winter and autumn (MK, tau: − 0.22 and − 0.21, respectively, *p* < 0.01 in both cases) and highly significantly in spring and summer (MK, tau: − 0.40 and MK, − 0.41, respectively, *p* < 0.001 in both cases; Supplement 1).

The cell size of the centric diatom community also decreased significantly, considering all seasons (Seasonal MK, tau: − 0.30, *p* < 0.001). In individual seasons (Figure 1B), the decreasing tendency was only significant in spring and summer (MK, tau: − 0.41 and − 0.41, *p* < 0.01 in both cases; Supplement 1).

The cell size of *Stephanodiscus* decreased significantly, considering the pooled data of all seasons (Seasonal MK, tau: − 0.28, *p* < 0.001). The decreasing tendency was only significant in winter and spring in individual seasons (MK, tau: − 0.37 and − 0.48, respectively, *p* < 0.01 in both cases; see Figure 1C and Supplement 1).

### The Assemblage-Level Cell Size Response of Phytoplankton to Environmental Changes

**Figure 2:**
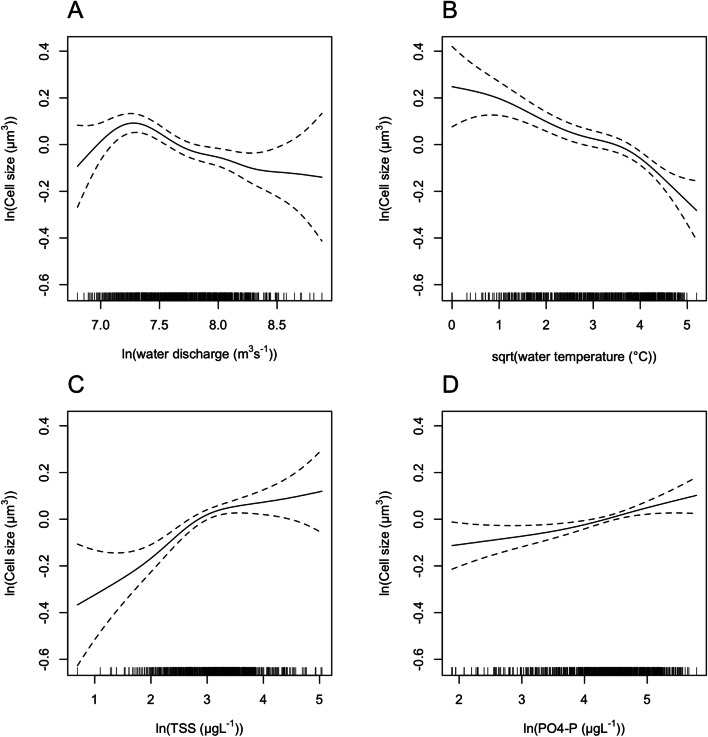
Relationship between the average cell size of phytoplankton and **A** water discharge; **B** water temperature; **C** total suspended solids (TSS); and **D** orthophosphate-P (PO4-P) in the middle Danube section (Göd, N-Budapest, Hungary) based on generalised additive model (GAM, *n* = 843, *R*_adj_^2^ = 0.209, *p* < 0.01 for PO4-P, *p* < 0.001 for all other predictors). Year and month (time) were set as random factors.

In the entire data set of the 34 years, environmental variables that affected the ACS of phytoplankton significantly were water discharge, water temperature, TSS and PO4-P (GAM, *R*_adj_^2^ = 0.209, *p* < 0.01 for PO4-P and *p* < 0.001 for all the other predictors). The ACS of phytoplankton decreased significantly with increasing water discharge and water temperature (Figure 2A, B), whereas it increased significantly with increasing concentration of total suspended solids (TSS) and orthophosphate-P (PO4-P) (Figure 2C, D).

### The Average Cell Size of Phytoplankton Affecting Planktic Algal Biomass

**Figure 3:**
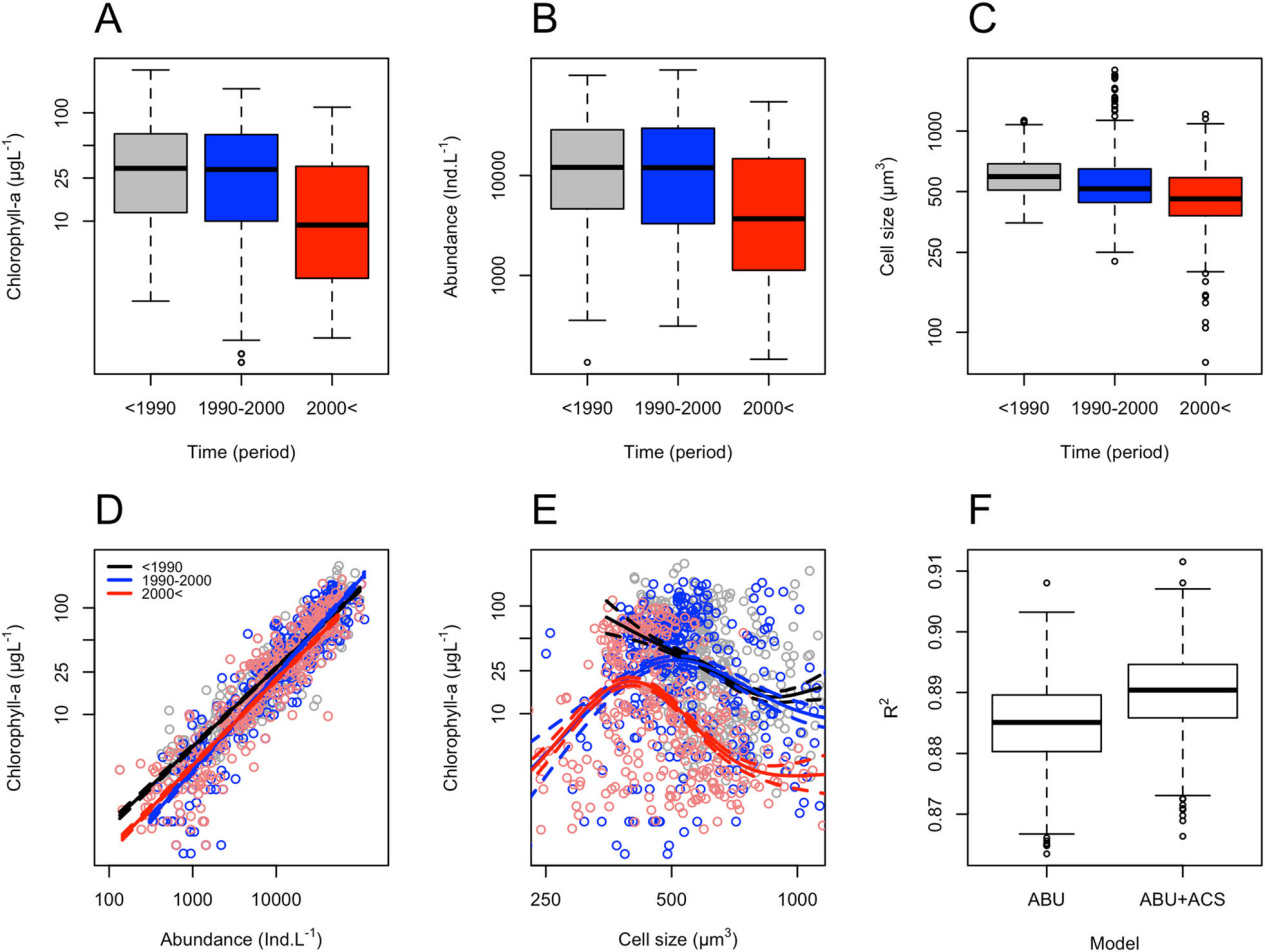
Differences between three discrete time periods (see Supplement 2): P1 (black): before 1990 (stable phase), P2 (blue): between 1990 and 2000 (transitional phase), and P3 (red): after 2000 (dispersed phase) in **A** boxplots of chlorophyll-*a*; **B** boxplots of phytoplankton abundance; **C** boxplots of average cell size of phytoplankton; **D** phytoplankton abundance predicting chlorophyll-*a* (LM, *R*_adj_^2^ = 0.8479 (P1), 0.8418 (P2), 0.8741 (P3), *p* < 0.001, in all cases); **E** average cell size of phytoplankton predicting chlorophyll-*a* (GAM, *R*_adj_^2^ = 0.137 (P1), 0.102 (P2), 0.283 (P3), respectively, *p* < 0.001, in all cases). **F** Boxplots of 999 bootstrapped coefficients of determination of generalised additive model (GAM) in predicting chlorophyll-*a* from algal abundance (ABU) and ABU+ average cell size (ACS) of phytoplankton (Wilcoxon, *p* < 0.001). Models are based on once a week phytoplankton samples from the middle Danube River, Göd (N-Budapest), Hungary (*n*_P1_ = 406, *n*_P2_ = 343, *n*_P3_ = 355) (Color figure online).

Based on the entire weekly data set, chlorophyll-*a*, algal abundance and average cell size of phytoplankton displayed three distinct periods (see Supplements 2). Although chlorophyll-*a* and algal abundance did not decrease before the 2000s (Figure 3A, B), average cell size of phytoplankton did so (Figure 3C). Independently of abundance (Figure 3D), the ACS of phytoplankton predicted chlorophyll-*a* in a distinct way between the three periods considered (Figure 3E):

1. Before 1990 (P1), median values of chlorophyll-*a* and algal density were high (> 25 µg L^−1^ and 10,000 ind. L^−1^, respectively, Figure 3A). The ACS of phytoplankton was relatively constant at about 500 µm^3^ (Figure 3C; stable phase). Chlorophyll-*a* correlated with algal abundance positively and significantly in a linear way (LM, Figure 3D), whereas it correlated linearly and negatively with the ACS of phytoplankton (GAM, Figure 3E);
2. Between 1990 and 2000 (P2), chlorophyll-*a* and phytoplankton abundance did not change significantly compared to P1 (Wilcoxon, *p* = 0.305 and 0.737, respectively), whereas the ACS of phytoplankton decreased significantly (Wilcoxon, *p* < 0.001; Figure 3C, transitional phase). Chlorophyll-*a* showed a sharp linear increase with algal abundance, but a broad hump-shaped relationship with the ACS of phytoplankton (Figure 3D, E).
3. After 2000 (P3), all the three parameters decreased significantly compared to P2 (Wilcoxon, *p* < 0.001, in all cases), and both small and large taxa occurred regularly in phytoplankton (Figure 3C; dispersed phase). Chlorophyll-*a* showed a steep linear increase with algal abundance and a narrow hump-shaped relationship with the ACS of phytoplankton (Figure 3D, E).

Based on the entire weekly data set, GAM predicted chlorophyll-*a* significantly better if the model also included the average cell size of phytoplankton on top of algal abundance. The ACS of phytoplankton increased the bootstrapped coefficients of determination (Figure 3F) and decreased AIC values significantly in GAMs (AIC_Δ_ = 41.3, Wilcoxon, *p* < 0.001, in both cases).

## Discussion

### The Cell Size Response of Phytoplankton to Environmental Changes

We hypothesised that phytoplankton cell size would decrease over time in the middle section of the Danube River at multiple organisation levels and also that environmental variables with long-term trends due to global change and human impacts would affect the average cell size of phytoplankton. Data evidenced the cell size decrease at multiple assemblage levels, coupled with a more dispersed cell size structure over time. Therefore, small and large individuals now both occur more often in the middle Danube.

Recent studies evidenced gradual water temperature increase (Moatar and Gailhard 2006; Abonyi and others 2018), alterations in the seasonality of water discharge (Hardenbicker and others 2014; Abonyi and others 2018), as well as nutrient declines (Minaudo and others 2015; Ibáñez and Peñuelas 2019) in large European rivers. Water temperature increase alone would be able to trigger the dominance increase in small-sized phytoplankton (Bopp and others 2005; Winder and others 2009). Water temperature increase, however, is mainly coupled with lower water discharge values that increase water retention time and decrease turbulence. Reduced turbulence enhances sedimentation that decreases turbidity (Tóth and Bódis 2015). Because sinking velocity of phytoplankton also depends on turbulent velocity of the water column (Reynolds 1997), enhanced sedimentation over time is also expected to constrain the Danube phytoplankton, especially large and “heavy” siliceous diatoms (Reynolds 1994).

Nutrient decline, observed mainly in PO4-P in the middle Danube (Istvánovics and Honti 2012; Abonyi and others 2018), triggers potentially the dominance of smaller-sized phytoplankton further. Smaller phytoplankton cell size means slower sedimentation (Sommer 1988) and represents higher surface-to-volume ratio, which enhances nutrient uptake under limiting conditions (Lewis 1976; Irwin and Oliver 2009; Finkel and others 2010). Accordingly, long-term cell size decrease in phytoplankton in the middle Danube may highlight the mechanism of long-term adaptation to altered environmental conditions. The mechanism, represented by the ACS decrease in phytoplankton in a meaningful way, however, can originate both from adaptation within the phenotypic plasticity range of individual taxa and from compositional change.

The phytoplankton of the middle Danube have primarily been composed by large-sized centric diatoms (Kiss 1994), well-adapted to turbulent, turbid and enriched conditions (Reynolds and Descy 1996). In response to increased underwater light availability, nutrient decline and the more frequent occurrence of low and extreme high flow events in the middle Danube, the functional diversity of phytoplankton increased over time (opt. cit.). This increase originated both from the occurrence of planktic taxa well-adapted to altered conditions (for example, small-sized and flagellated taxa) and from dispersed benthic and limnophilic elements. The composition of phytoplankton also shifted gradually over time. The dominance of large-sized eutrophic centric diatoms decreased (for example, *Stephanodiscus hantzschii* and *S. hantzschii* var. *tenuis*, core taxa in the middle Danube River), whereas small-sized and dispersed elements increased. Accordingly, both community shift and individual adaptations of taxa are part of the long-term response of the middle Danube phytoplankton to altered conditions.

Because both smaller phytoplankton taxa and dispersed elements now occur more often in the middle Danube, one may expect that the ecosystem functioning of phytoplankton has also altered over time. In this case, the relationship between algal biomass production (chlorophyll-*a*) and the average cell size of phytoplankton should have altered over time.

### The Altered Cell Size Structure of Phytoplankton Constrains Ecosystem Functioning

We expected that the altered cell size structure of phytoplankton would affect the relationship between ecosystem functioning (chlorophyll-*a* production) and the average cell size of phytoplankton over time. Our results supported this expectation, and the relationship shifted from negative linear towards a broad and then a narrow hump shape one over time.

Turbulent and turbid large rivers represent a highly selective environment (Reynolds and others 1994), often resulting in the dominance of large, eutrophic centric diatoms (Reynolds and Descy 1996) in low diversity assemblages (Margalef 1978). Middle- and large-sized individuals contribute to biomass production in a highly efficient way (Marañón 2015). The fact that the middle Danube phytoplankton was dominated by large- and medium-sized centrics before the 1990s, high algal abundance (bloom conditions) resulted in high planktic algal biomass and therefore in a highly efficient ecosystem functioning. The negative linear relationship between chlorophyll-*a* and the ACS of phytoplankton may represent the deterministic process of centric diatoms’ growth under favourable conditions. Although one may expect the dominance of larger-sized taxa in high flow conditions, stable turbulent and turbid high flow coupled with high resource availability may lead to the enhanced growth of centric diatoms, that is, the accelerated rate in valve multiplication leading to cell size decrease (Jewson 1992).

With time, both the dominance and cell size of large, eutrophic diatoms decreased, which independently of algal abundance, could lead to chlorophyll-*a* decrease in the middle Danube between 1990 and 2000 (P2). Productive, high chlorophyll-*a* phytoplankton appeared in a rather constant cell size range (~ 450–500 μm^3^) in our data set, independently of the period considered. Accordingly, the long-term decrease in cell size of centric diatoms presumes alone decrease in the ecosystem functioning of phytoplankton. The long-term shift from a linear to a hump-shaped relationship between chlorophyll-*a* and the ACS of phytoplankton, therefore, may require the consideration of further mechanisms.

River phytoplankton tends to be more light-limited than lake phytoplankton (Reynolds and others 1994). The enhanced sedimentation of suspended solids resulted in high underwater light availability, especially in late summer (Vörös and others 2000). This, coupled with high water temperature and nutrient decrease, may have altered the environment beyond the phenotypic plasticity of certain diatoms, especially of shade-adapted large eutrophic taxa. Decrease in ecosystem functioning in the smaller size range, therefore, may also be due to the dominance increase in better adapted taxa to low flow conditions like flagellates (for example, *Plagioselmis*, *Chroomonas*), or to high light availability like *Skeletonema potamos* (Kiss and others 2012; Duleba and others 2014).

Due to the more frequent occurrence of extreme high flow conditions, dispersed limnophilic and benthic taxa now occur more frequently in the middle Danube plankton (Abonyi and others 2018). Although the majority of these taxa are large that increase the ACS of phytoplankton, they are non-adapted to river conditions and do not compete for resources in an efficient way. Accordingly, these “passive” dispersed elements do not contribute to planktic algal biomass to a large extent. Consequently, coupled with the dominance decrease in large-sized diatoms in the middle Danube, the more frequent occurrence of dispersed limnophilic and benthic taxa predicts also low ecosystem functioning (chlorophyll-*a*) in the larger range of the ACS of phytoplankton.

## Conclusions

Environmental changes coupled to global warming and human impacts altered the cell size structure of phytoplankton in the middle section of the Danube River. Cell size of phytoplankton decreased over time in average values within assemblages and also within the best-adapted and therefore the most productive taxonomic group in large rivers: centric diatoms. Due to the highly selective environment, large river phytoplankton constitutes mainly low diversity assemblages under the dominance of centric diatoms. The long-term decrease in phytoplankton cell size, especially within centric diatoms, may highlight that large river phytoplankton is vulnerable to global change and human impacts at the long temporal scale.

Long-term decrease in phytoplankton cell size and the altered cell size structure constrained planktic algal biomass production in the middle Danube and expectedly in other large rivers. Body size is coupled to metabolic constraints (opt. cit.) and affects food web functioning fundamentally (Woodward and others 2005). The long-term cell size decrease in large river primary producers may, therefore, constrain higher trophic levels further. Such a cascading effect could enhance the more frequent occurrence of low production, “clear-water” plankton in large rivers under multiple pressures from human impacts and global environmental change.

## Electronic supplementary material

Below is the link to the electronic supplementary material.

**Supplement 1.** Mann–Kendall trend analysis of cell size (biovolume) based on (A) the average cell size (ACS) of phytoplankton; (B) the entire centric diatom community; (C) the Stephanodiscaceae–Thalassiosiraceae families in the middle Danube section, Göd (N-Budapest, Hungary) between 1979 and 2012. Tau and significance levels (in brackets) are given, as well as bootstrap confidence interval calculations based on 10,000 bootstrap replicates at 99% CI (in the second line). Significant trends are bold and italic. N.s.: non-significant, *: *p* < 0.05, **: *p* < 0.01, ***: *p* < 0.001. (DOCX 45 kb)

**Supplement 2.** Chlorophyll-*a* in the Danube River as a function of the average cell size of phytoplankton (total algal biovolume to the total algal abundance ratio) and time. The relationship shows three discrete periods: (1) before 1990—stable phase; (2) between 1990 and 2000—transitional phase; and (3) after 2000—dispersed phase. The Figure is based on once a week sampling frequency from the middle Danube section, Göd (N-Budapest) Hungary, from the period 1979 to 2012 (*n* = 1434). (PNG 211 kb)

**Supplement 3.** AAbonyi_Supplement3_Ecosystems_2019: Quantitative data of the Danube River phytoplankton and environmental variables (1979-2012). (CSV 132 kb)

**Supplement 4.** AAbonyi_Supplement4_MedianCentrales_Duna: Quantitative data of median cell size values of the centric diatom community in the middle Danube River (1979-2012). (CSV 3 kb)

**Supplement 5.** AAbonyi_Supplement5_MedianStephano_Duna: Quantitative data of median cell size values of *Stephanodiscus* in the middle Danube River (1979-2012). (CSV 3 kb)

Supplementary material 6 (DOCX 12 kb)
