# Supplementary material for: Cell Size Decrease and Altered Size Structure of Phytoplankton Constrain Ecosystem Functioning in the Middle Danube River Over Multiple Decades
Source: Ecosystems. 2019 Dec 3;23(6):1254–64. doi: 10.1007/s10021-019-00467-6 (PMC7497449; doi:10.1007/s10021-019-00467-6)
Supplement: Supplementary file 1 — Supplement 1. Mann–Kendall trend analysis of cell size (biovolume) based on (A) the average cell size (ACS) of phytoplankton; (B) the entire centric diatom community; (C) the Stephanodiscaceae–Thalassiosiraceae families in the middle Danube section, Göd (N-Budapest, Hungary) between 1979 and 2012. Tau and significance levels (in brackets) are given, as well as bootstrap confidence interval calculations based on 10,000 bootstrap replicates at 99% CI (in the second line). Significant trends are bold and italic. N.s.: non-significant, *: p < 0.05, **: p < 0.01, ***: p < 0.001. (DOCX 45 kb) [file 10021_2019_467_MOESM1_ESM.docx]

| **Level of organization** | **WINTER**  (Mann-K) | **SPRING**  (Mann-K) | **SUMMER**  (Mann-K) | **AUTUMN**  (Mann-K) |
| --- | --- | --- | --- | --- |
| **A) Average cell size of phytoplankton** | ***-0.22 (**)***  ***(-0.78, -0.12)*** | ***-0.40 (***)***  ***(-1.11, -0.49)*** | ***-0.41 (***)***  ***(-1.15, -0.49)*** | ***-0.21 (**)***  ***(-0.72, -0.12)*** |
| **B) Centric diatom community** | -0.21 (N.s.)  (-0.96, -0.04) | ***-0.41 (**)***  ***(-1.29, -0.33)*** | ***-0.41 (**)***  ***(-1.29, -0.32)*** | -0.12 (N.s.)  (-0.60, 0.13) |
| **C) Stephanodiscaceae – Thalassiosiraceae families** | ***-0.37 (*)***  ***(-1.28, -0.19)*** | ***-0.48 (*)***  ***(-1.40, -0.52)*** | -0.08 (N.s.)  (-0.55, 0.20) | -0.27 (N.s.)  -0.96, -0.08 |
