# Supplementary figures and images for: Cell Size Decrease and Altered Size Structure of Phytoplankton Constrain Ecosystem Functioning in the Middle Danube River Over Multiple Decades
Source: Ecosystems. 2019 Dec 3;23(6):1254–64. doi: 10.1007/s10021-019-00467-6 (PMC7497449; doi:10.1007/s10021-019-00467-6)

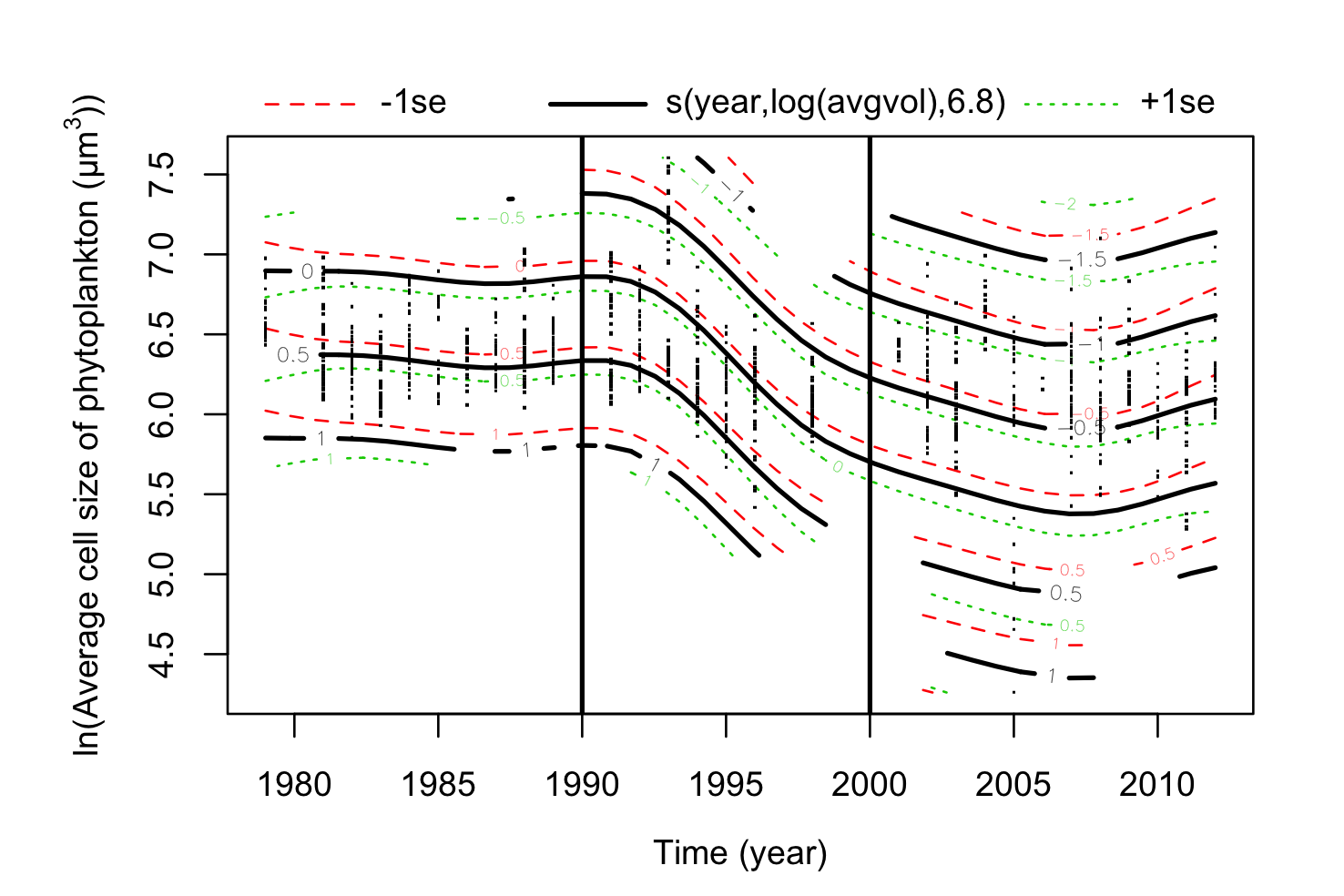

Supplement: Supplementary file 2 — Supplement 2. Chlorophyll-a in the Danube River as a function of the average cell size of phytoplankton (total algal biovolume to the total algal abundance ratio) and time. The relationship shows three discrete periods: (1) before 1990—stable phase; (2) between 1990 and 2000—transitional phase; and (3) after 2000—dispersed phase. The Figure is based on once a week sampling frequency from the middle Danube section, Göd (N-Budapest) Hungary, from the period 1979 to 2012 (n = 1434). (PNG 211 kb) [file 10021_2019_467_MOESM2_ESM.png]
