# Supplementary material for: Cell Size Decrease and Altered Size Structure of Phytoplankton Constrain Ecosystem Functioning in the Middle Danube River Over Multiple Decades
Source: Ecosystems. 2019 Dec 3;23(6):1254–64. doi: 10.1007/s10021-019-00467-6 (PMC7497449; doi:10.1007/s10021-019-00467-6)
Supplement: Supplementary file 6 — Supplementary material 6 (DOCX 12 kb) [file 10021_2019_467_MOESM6_ESM.docx]

ECOSYSTEMS MANUSCRIPT INFORMATION SHEET

MANUSCRIPT NUMBER: ECO-19-0249.R2

TITLE: Cell size decrease and altered size structure of phytoplankton constrain ecosystem functioning in the middle Danube River over multiple decades

AUTHORS: Abonyi, András; Kiss, Keve; Hidas, András; Borics, Gábor; Várbíró, Gábor; Ács, Éva

CORRESPONDING AUTHOR:

Dr. András Abonyi

Institute of Ecology and Botany

MTA Centre for Ecology

Alkotmány u 2-4

Lunz am See 2163

Hungary

FAX:

PHONE:

EMAIL: [abonyi.andras@okologia.mta.hu](mailto:abonyi.andras@okologia.mta.hu)

RECEIVED 25-Jul-2019; ACCEPTED 13 NOVEMBER 2019

COLOR FIGURES: 2

Author agrees to pay charges

COMMENTS:
